# The Bromides in Dentistry

**Published:** 1884-11

**Authors:** R. M. Sanger


### THE BROMIDES IN DENTISTRY.

BY R. M. SANGER, D. D. S.

READ BEFORE THE NEW JERSEY STATE DENTAL SOCIETY AT ITS FOURTEENTH ANNUAL MEETING, ASBURY PARK, JULY, 1884.

*Mr. President and Gentlemen:*

The subject which I have chosen for my paper is not exclusively dental, and my excuse for offering it must be that since it is a large part of the dentist's mission to relieve human suffering, everything which aids in any way that end, or comes in as an adjuvant in accomplishing that purpose, should be welcome to the dentist and be found among his varied resources.

The peculiar effects of the Bromides, as seen in medical practice, suggest several therapeutic uses in dentistry, and on these and the results of their use in my own practice I base my claim to bring them before you. Though the effects of the Bromides are doubtless well known to you all, it will serve my purpose and make my subject clearer to briefly review their action and bring out a few of the salient points. While they differ slightly in their action and effect, I take the Bromide of Potassium as fairly representative of the group, and the one most commonly used.

The minor points of solubility, taste, etc., I will pass over, simply remarking that in consequence of its ready solubility in water it is easily administered, while the taste is simply saline, and not highly disagreeable to any one.

It is more of its physiological effect that I wish to speak, and just here I will remark that we gain no aid in studying the effect of this medicine from its administration to the lower animals, as the results of a medicinal dose are very misleading, being to them a poison, while to man it is much milder, not a single case of acute poisoning having ever occurred. It is true that in large doses, long continued, certain effects are produced which might be classed as poisonous, but with these we have nothing to do, as my object is to show the use which can be made of a single dose. The immediate effects, physiologically considered, are:

1st. Depression of the heart's action.

2d. Diminished respiration.

3d. Lowering of the temperature of the body.

Bartholow's observations are that "two drams of Bromide of Potassium will lower the temperature in a healthy adult from one-fifth to one-half a degree, the respiration from two to five, and the pulse from ten to twenty beats per minute. The sensibility to pain, but especially the sensibility to tactile impressions, is lowered by the Bromides at all accessible points of the mucous membrane and the skin. The diminution of the sensibility of the mucous membrane is due in part to a local action of the salt as it is being eliminated. They also possess the power to destroy or impair the irritability of the motor and sensory nerves, and the contractility of muscle."

Now I can state categorically the therapeutic uses which may be made of Bromide of Potassium in dentistry, and we can all see and understand the *modus operandi.*

1st. We can use Bromide of Potassium to quiet cerebral excitement.

2d. We can use it to diminish the sensibility to tactile impressions in the mouth.

4th. We can administer it freely without any fear of unpleasant results.

Our brethren in the medical profession are demonstrating the value of the Bromides in these respects every day, and that not in serious maladies alone, but in the more trivial yet none the less disagreeable, just such as we meet every day in our own practice.

The physician is called in to see a patient suffering from severe mental excitement, caused, perhaps, by nothing more than a "scene" in the family. There is no disease, but simply that condition of cerebral excitement and reflex irritability vulgarly called "hysterics." Our clear headed physician perceives the condition, administers a full dose of Bromide of Potassium, and soon the peculiar effects are seen. The brake is applied to undue excitement and irritability, and a condition of peace and quiet follows.

The novice in public speaking having the ordeal of an address or lecture before him, knows by certain preliminary signs, and perhaps by past experience, that when he shall stand before his audience in a state of high mental excitement the follicles of the mouth and pharynx will close and leave the tongue cleaving to the roof of the mouth, the throat suggestive of a very dry spell of weather, the heart beating an alarm, a "globus hystericus" in the throat (the lump which can be swallowed but not kept down), the air passages so occluded in consequence of the nervous excitement that 100 breaths a minute seem a necessity, the hands and feet unable to find a resting place, and, more than all, the very opening sentence of a carefully prepared and well memorized address completely gone from memory. Now thus forewarned he goes to a physician and states the case. The one who is not familiar with the virtue of the Bromides may laugh and joke, but the one who does know orders thirty grains of the Bromide of Potassium, to be taken one-half hour before the time of need. And with this brake on nervous excitement the speaker comes up to the ordeal smiling, fresh and cool as a veteran.

The *Laryngoscopist* finds a patient who, the moment a mirror is placed in his throat, so far forgets himself as to try to swallow it. The examination of the larynx cannot be made. What is to be done? The trouble is simply reflex. The patient has the best intentions, but is helpless. Scolding is of no use. The physician simply orders a gargle of Potassium Bromide to be used frequently, and directs the patient to come again the next day. Then there is no trouble. The brake has been applied; the patient cannot be made to swallow the mirror, and the examination is completed with perfect ease.

And now I come to the practical part of my subject, to which these remarks have been leading. Certainly the results which the physician obtains from Bromide of Potassium in his practice, in these trivial matters, as some may be inclined to call them, are highly suggestive to us as dentists, and may be applied in somewhat similar cases in our own practice, cases which, if not serious, are very trying to both patient and operator. I refer to all that class of troubles we meet characterized by cerebral excitement, heightened reflex irritability, or marked by hyper-sensitiveness to tactile impressions.

It is scarcely necessary for me to say that a perfect fitting denture cannot be made without a perfect impression, and yet how often we try to obtain a satisfactory result with one we know to be imperfect, because it seems impossible to get a better, since each attempt produces a paroxysm of coughing and choking on the part of the patient that compels us to withdraw the cup sooner than we wish. If this condition were due to foreign matter in the pharynx or larynx, actually impeding respiration, we should have no remedy; but since it is so often produced when that condition does not exist, it can be classed only in the same category of reflex disturbances that the *Laryngoscopist* meets, and this being the case thirty grains of Bromide of Potassium given immediately on the arrival of the patient will, one-half hour later, obviate this difficulty and render the patient as docile in our hands as in the hands of our medical friend.

Again; a patient presents herself desiring to have gas administered, but so nervous that she can scarcely sit still while you are making the examination, and sometimes with a doleful tale of how she almost "cleaned out" another dentist's office when he attempted to administer the anæsthetic, and he was compelled to perform the operation while she was only partially unconscious, thereby causing more mental suffering than if the gas had been dispensed with. Now the trouble is increased by the memory of that occasion. But it can all be remedied and the way made clear by the administration of from thirty to forty-five grains of Bromide of Potassium.

Again; how frequently we have patients who tell us that they dread the after effects of a sitting far more than the actual pain of the operation, as they invariably suffer from nervous prostration and headache for hours. Administer thirty grains of Bromide of Potassium about one-half hour before you begin, and the patient will leave your chair after a sitting of an hour or more surprised to find himself as well as when the operation commenced. Then we can take a hint from the fact that Bromide of Potassium will lessen the sensibility to tactile impressions, and give a dose freely when we have a very sensitive tooth to deal with. The result I have found to be perfectly in accordance with what we should expect.

